# The World Health Organization Access, Watch, and Reserve classification of antibiotics: an awareness survey among pharmacy professionals in a sub-Saharan country, Zambia

**DOI:** 10.1017/ash.2024.403

**Published:** 2024-12-02

**Authors:** Steward Mudenda, McDonald David Wataya, Webrod Mufwambi, Joseph Yamweka Chizimu

**Affiliations:** 1 Department of Pharmacy, School of Health Sciences, University of Zambia, Lusaka, Zambia; 2 Antimicrobial Resistance Coordinating Committee (AMRCC), Zambia National Public Health Institute, Lusaka, Zambia; 3 African Global Logistics, Blantyre, Malawi

## Abstract

**Background:**

Antimicrobial stewardship programs are very essential in addressing the problem of drug-resistant infections. The WHO Access, Watch, and Reserve (AWaRe) classification of antibiotics is essential in monitoring the rational use of antibiotics. Therefore, this study evaluated the awareness of the WHO AWaRe classification of antibiotics among pharmacy professionals in Zambia.

**Materials and methods:**

This cross-sectional study was conducted among 239 pharmacy professionals practicing in both private and public facilities in Zambia. A questionnaire was used to collect data which was subsequently analyzed using IBM SPSS version 23.0.

**Results:**

Of the 239 participants, 63% were male and most were pharmacists employed in the public sector. This study found that 58% of the pharmacy professionals were aware of the AWaRe classification of antibiotics. Consequently, only 36% of the participants agreed that they implemented the AWaRe framework of antibiotics in their healthcare facilities. Most of the participants (74%) agreed that implementing the AWaRe tool can promote the rational use of antibiotics and 98% of the participants were willing to learn more about the AWaRe tool through training or meetings.

**Conclusion:**

This study found a low awareness of the WHO AWaRe classification of antibiotics among pharmacy professionals. Our study further revealed that very few pharmacy professionals agreed that they had implemented the WHO AWaRe tool in their healthcare facilities. Hence, there is a need to strengthen antimicrobial stewardship programs by implementing the AWaRe framework and other recommended guidelines for monitoring the rational use of antibiotics.

## Background

Antimicrobial stewardship (AMS) programs were instigated and implemented to address the global public health problem of antimicrobial resistance (AMR).^1–3^ AMR occurs when microorganisms stop responding to antimicrobials to which they were previously susceptible to leading to challenges in treating infections.^4^ AMR is a global public health problem with many consequences including increased morbidity and mortality, medical costs, and negative impact on the global economy.^5^ If this trend persists, many lives will be lost, with an estimated 10 million deaths annually by the year 2050.^5^ Therefore, there is a need to implement and strengthen antimicrobial stewardship programs to address AMR and reduce its consequences.^6,7^

AMS programs are instigated to optimize the use of antimicrobials, improve patient outcomes, reduce medical costs, and curb AMR.^2,3^ These programs further promote awareness of AMR among healthcare workers and the general population.^2^ Antimicrobial stewardship programs also promote adherence to treatment guidelines to foster rational use of antimicrobials.^8^ Therefore, through antimicrobial stewardship programs, the right antimicrobials must be prescribed for the right patient, for the right diagnosis, at the right time, with the right dose, right route of administration, and duration.^1,9,10^

In the year 2015, the WHO developed the Global Action Plan (GAP) on AMR with a vision to address the problem of drug resistance.^11^ The WHO, FAO, WOAH, and UN recommends countries to develop and implement their National Action Plans (NAP) on AMR.^11^ The GAP and NAPs on AMR promote awareness and knowledge concerning AMR, strengthen surveillance and research regarding AMR, reduce the incidence of infections, optimize the use of antimicrobials, improve investment in the development of new medicines, vaccines, and diagnostic tools, and help instigate strategies to address AMR using a One Health approach.^11^ Some countries have already developed and implemented their NAP on AMR in line with the GAP and are addressing AMR in humans, animals, agriculture, and the environment.^12^

In the year 2017, the WHO developed the Access, Watch, and Reserve (AWaRe) classification of antibiotics as a framework to monitor the consumption of antibiotics, their appropriate use, and the impacts of antimicrobial stewardship programs in addressing the global problem of AMR.^13,14^ The Access group antibiotics include narrow-spectrum antibiotics that are recommended as first and second-line empiric treatment of the commonest clinical syndromes.^15,16^ The Watch group antibiotics generally include broad-spectrum antibiotics which are critically important for human medicine but have a higher potential to develop AMR and thus should be used for critically ill patients in hospital settings.^15,16^ Further, the Reserve group antibiotics include antibiotics that are used as a last resort and reserved for multidrug-resistant pathogens.^16,17^ The AWaRe framework was established to promote the rational prescribing and use of antibiotics and reduce the emergence and spread of AMR.^13,14,18^ Based on this framework, it was estimated that by 2023, each healthcare facility was to prescribe at least 60% of Access group antibiotics.^14,17,19^

Zambia developed its NAP on AMR in the year 2017 and implemented it through the Antimicrobial Resistance Coordinating Committee (AMRCC) of the Zambia National Public Health Institute.^20^ Furthermore, Zambia has embraced the WHO AWaRe classification and is implementing it in certain healthcare facilities through the AMRCC, which leads the effort.^21^ Some studies have reported irrational prescribing by not adhering to the WHO AWaRe classification of antibiotics among prescribers in Zambia.^22–25^ Additionally, resistance of pathogens to antimicrobials has been reported.^26,27^ However, there is no information or studies that have been published on the awareness of the WHO AWaRe classification of antibiotics among healthcare workers. Therefore, this study was conducted to fill this gap and evaluate the awareness of the WHO AWaRe classification of antibiotics among pharmacy professionals working in private and public healthcare facilities in Zambia.

## Materials and methods

### Study design, site, and population

A cross-sectional study was conducted from February 2024 to March 2024 among pharmacy professionals in Zambia. To be eligible, every pharmacy professional was to be registered with the Health Professions Council of Zambia as a pharmacist or pharmacy technologist. Additionally, only pharmacy professionals who provided informed and written consent to participate in the study were enrolled in this survey.

### Sample size estimation

We used Taro Yamane’s formula to estimate the sample size.^28^ With no previous study done on the awareness of the WHO AWaRe classification of antibiotics, we used a population of 1025 pharmacy professionals to determine the sample size at a 95% confidence level and a margin error of 5%. We obtained a minimum sample size of 288 to be used in the study. Since it was not possible to meet all the pharmacy professionals physically across the country, we shared a Link containing the Google Form (questionnaire) and consent form in the WhatsApp group that contained 1025 pharmacy professionals. All participants who met the inclusion criteria were requested to participate in this study. We stratified the pharmacy professionals into pharmacists and pharmacy technologists. The estimated sample size was not met due to non-response from the target population.

### Data collection

Data collection was conducted using a structured questionnaire. The questionnaire was developed and reviewed by pharmacists and clinicians who practice in policymaking, hospitals, and academia. The questionnaire was reviewed for accuracy, simplicity, understandability, clarity, and relevance by expert pharmacists in the field of public health. Face and content validation of the questionnaire was done by pharmacists working in academia and the Ministry of Health, Zambia. The questionnaire was piloted among 10 pharmacists practicing in the public health sector. The questionnaire had eight questions including three on sociodemographics and five questions on awareness and use of the WHO AWaRe classification of antibiotics. A question on awareness had two responses, i.e. “Yes” or “No.” Each participant took five to ten minutes to complete filling in the questionnaire. Data collection was done by three pharmacists who are involved in the fight against AMR in Zambia.

### Data analysis

The collected data were entered in Microsoft Excel sheet version 2013 for validation and exported to IBM SPSS version 23.0 for analysis. Like other studies done on awareness and knowledge, being aware of the WHO AWaRe classification of antibiotics was assigned a percentage score of 80% and above.^29–31^ A “Yes” response was assigned a score of 1 while a “No” or I don’t know response was assigned a score of 0. Hence, each question was scored out of 100% by dividing the number of correct responses by the total responses and multiplying by 100. All the findings were presented in Tables. Univariate analysis was performed to determine factors that influenced awareness and use of the AWaRe classification of antibiotics. Statistical significance was set at p<0.05.

### Ethical approval

We obtained ethical approval from the Tropical Diseases Research Centre Ethics Committee with an approval number of TRC/C4/09/2023. The data collectors informed the participants of the study objectives. All participants were informed that participation was voluntary purpose. Participation in the study was only possible after providing informed consent and ticking on “accept to participate in this study.”

## Results

### Sociodemographics of study participants

This study enrolled 239 pharmacy professionals giving a response rate of 83%. Overall, 63% were female, 68% were pharmacists, 32% were pharmacy technologists, and most participants were employed in the public sector (51%) (Table 1).

**Table 1.** Sociodemographic characteristics of participants

Gender	Frequency	Percent
Female	88	37
Male	151	63
**Profession**		
Pharmacist	163	68
Pharmacy Technologist	76	32
**Employment Status**		
Employed in Public Sector	121	51
Employed in Private Sector	73	30
Not employed	45	19

This study found that 58% of the pharmacy professionals were aware of the WHO AWaRe classification of antibiotics (Table 2). Further, 36% of the pharmacy professionals responded that they had implemented the WHO AWaRe classification of antibiotics in their facilities (Table 2). Furthermore, 74% of the pharmacy professionals responded that implementation of the WHO AWaRe classification of antibiotics would promote the rational use of antibiotics (Table 2). The present study found that 70% of the pharmacy professionals responded that the use of the WHO AWaRe classification of antibiotics is a game changer in antimicrobial stewardship (Table 2). Notably, 98% of the pharmacy professionals were willing to learn more about the WHO AWaRe classification of antibiotics (Table 2).

**Table 2.** Participants’ responses regarding their awareness, use, and willingness to learn about the WHO AWaRe classification of antibiotics

Statement	Response	Frequency	Percent
I am aware of the WHO AWaRe classification of antibiotics	Yes	139	58
	No	100	42
The AWaRe classification of antibiotics is used at our facility	Yes	85	36
	No	154	64
The AWaRe framework promotes rational use of antibiotics	Yes	176	74
	No	63	26
The AWaRe framework is a game changer in the fight against AMR	Yes	167	70
	No	72	30
I am willing to learn more about the AWaRe classification of antibiotics	Yes	234	98
	No	5	2

This study found that there was no relationship between awareness of the WHO AWaRe classification of antibiotics and the sociodemographic characteristics of participants (Table 3). However, it was observed that most male pharmacists employed in the public sector were aware of the AWaRe classification of antibiotics compared to females, pharmacy technologists, and those employed in the private sector (Table 3). This study revealed that most male pharmacists employed in the public sector confirmed that the AWaRe classification of antibiotics was used in their healthcare facilities compared to females, pharmacy technologists, and those employed in the private sector (Table 3).

**Table 3.** Factors influencing awareness and use of the Access, Watch, and Reserve (AwaRe) classification of antibiotics

		Awareness of the tool			Use of AWaRe tool		
Characteristic		Yes	No	p-value	Yes	No	p-value
**Gender**	Female	46	42	0.175	27	61	0.004
	Male	93	58		58	93	
**Profession**	Pharmacist	98	65	0.400	55	108	0.279
	Pharmacy Technologist	41	35		30	46	
**Employment Status**	Employed in Public Sector	71	50	0.907	47	74	0.001
	Employed in Private Sector	41	32		25	48	
	Not employed	27	18		13	32	

## Discussion

To the best of our knowledge, this was the first study to be conducted on the awareness of the WHO AWaRe classification of antibiotics among pharmacy professionals in Zambia. This study found that 58% of the participants were aware of the WHO AWaRe classification of antibiotics. However, only 36% responded that they implemented the use of the AWaRe classification of antibiotics in their facilities.

Our study found a low awareness of the AWaRe classification of antibiotics among pharmacy professionals. Our findings are higher than those that were reported in Jordan where only 21.5% of healthcare workers were aware of the WHO AWaRe classification of antibiotics pre-antimicrobial stewardship intervention stage.^32^ A study that was conducted in India found that only 27.7% of dental surgeons were aware of the WHO AWaRe classification of antibiotics indicating that the majority, 72.3%, were not aware of this important framework for addressing AMR.^33^ Another Indian study found that 87.2% of dental surgeons were not aware of the WHO AWaRe classification of antibiotics, indicating a low awareness of 22.8%.^34^ Additionally, our study found a higher awareness of the WHO AWaRe classification of antibiotics compared to a study that was done in India where more than 50% of physicians were not aware of this framework of antibiotics.^35^ This low awareness regarding the WHO AWaRe classification of antibiotics reported in our study and other studies could be due to limited and small-scale AMR awareness campaigns conducted in many countries.^36^

The low awareness of the WHO AWaRe classification of antibiotics in our study and similar studies demonstrate the need to strengthen antimicrobial stewardship interventions as they have been proven to improve awareness, knowledge, and rational use of antibiotics.^32,37^ A lack of awareness of the AWaRe classification of antibiotics can potentially lead to the overuse and misuse of antibiotics.^33^ Consequently, a lack of awareness of the AWaRe classification of antibiotics has led to the overuse of Watch group antibiotics which have a high potential to develop resistance.^38^ To address this low awareness, there is a need to instigate and strengthen antimicrobial stewardship programs in all hospitals. For instance, in Jordan, the implementation of antimicrobial stewardship interventions led to an improvement in the awareness of healthcare workers concerning the AWaRe classification of antibiotics from 21.5% to 58.5%, indicating the importance of instituting and implementing antimicrobial stewardship programs in healthcare facilities.^32^

Our study found that only 36% of the pharmacy professionals agreed that the use of the AWaRe classification of antibiotics has been implemented in their facilities. This score revealed a very low adaptation and use of the AWaRe tool in Zambia and thus may lead to inappropriate prescribing of antibiotics, as reported in earlier studies.^22,23^ Our study revealed that the majority of the pharmacy professionals agreed that the AWaRe classification of antibiotics is a game changer in the fight against AMR and were willing to learn more about it through meetings and training. This indicated an opportunity for the antimicrobial stewardship implementers to conduct training and the importance of adhering to the WHO AWaRe classification of antibiotics. This can be achieved through instigating educational initiatives across healthcare workers.^39,40^ This would eventually address the GAP’s and NAP’s objectives of increasing awareness and knowledge to address AMR.^11,20^

We are aware that our study has limitations. Firstly, it was a cross-sectional study, thereby, it cannot be used to analyze the behavioral change over time. Secondly, it cannot detailed information regarding antimicrobial stewardship campaigns and awareness levels of AWaRe classification of antibiotics. Thirdly, our sample size may not be representative of all pharmacists and pharmacy technologists in Zambia, hence, generalization of the findings must be done with caution. However, our findings are very encouraging because the identified awareness gaps can be used to strengthen education and training on the AWaRe classification of antibiotics. The present study also demonstrated the need to provide educational interventions regarding the WHO AWaRe classification of antibiotics to pharmacy professionals working in the private sector. Additionally, our findings are very instrumental in developing educational interventions targeted at healthcare workers regarding antibiotic use guidelines, awareness, and successful implementation of the WHO AWaRe classification of antibiotics as a tool for antimicrobial stewardship and promoting rational use of antibiotics.

## Conclusion

The study found a low awareness and use of the WHO AWaRe classification of antibiotics among pharmacy professionals in Zambia. However, most pharmacy professionals were willing to be educated and trained about the AWaRe classification of antibiotics. Therefore, there is a need to strengthen antimicrobial stewardship programs regarding the implementation of the AWaRe classification of antibiotics and other recommended guidelines for monitoring and promoting the rational use of antibiotics.

## Data Availability

Data can be made available on request from the corresponding author.
